# Comparative Genome and Transcriptome Study of the Gene Expression Difference Between Pathogenic and Environmental Strains of *Prototheca zopfii*

**DOI:** 10.3389/fmicb.2019.00443

**Published:** 2019-03-07

**Authors:** Xuanhao Zeng, Timothy Kudinha, Fanrong Kong, Qiang-qiang Zhang

**Affiliations:** ^1^Division of Mycology, Huashan Hospital, Fudan University, Shanghai, China; ^2^Charles Sturt University, Leeds Parade, Orange, NSW, Australia; ^3^Centre for Infectious Diseases and Microbiology Laboratory Services, ICPMR-Pathology West, Westmead Hospital, The University of Sydney, Sydney, NSW, Australia

**Keywords:** *Prototheca zopfii*, protothecosis, genome, transcriptome, virulence

## Abstract

*Prototheca zopfii* commonly exists in the environment, and causes invasive infections (protothecosis) in humans. The morbidity of protothecosis has increased rapidly in recent years, especially in systemic infections of patients with an impaired immune system. The infection in immunocompromised patients has a poor prognosis due to limited understanding of the pathogenesis of the disease, as most previous studies mainly focused on classification and recognition of pathogenic strains. In this study, we constructed the genome and transcriptome of two pathogenic strains and one environmental strain, by next generation sequencing methods. Based on our preliminary gene expression findings, genes in *P. zopfii* pathogenic strains are significantly up-regulated in metabolism in peroxisome, such as glyoxylate cycle, which may improve the organism’s resistance to the harsh environment in phagolysosome of macrophage and its ability to survive in an anaerobic environment. We also found some significant up-regulated genes, which are related to adherence and penetration in dermatophytes, and we speculate that this may enhance the virulence capacity of pathogenic strains. Finally, the genomes and transcriptomes of *P. zopfii* described here provide some base for further studies on the pathogenesis of this organism.

## Introduction

The taxonomy of *Prototheca* has been controversial for some time, but is currently classified among achlorophyllic algae, the *Chlorophyceae*, and is widely spread in the environment. *Prototheca* is the only human pathogen that is related to algae (Kwiecinski, 2015). Currently, six *Prototheca* species have been described, five of which have been proven to cause human infections. Worldwide, cases of protothecosis are rare. However, a rise in cases of protothecosis has been reported in recent years, indicating an increasing morbidity of this disease, especially in patients with impaired immune systems (Todd et al., 2012). In addition, it is difficult to diagnose protothecosis due to limited current knowledge on the organism, resulting in many patients failing to get timely and appropriate treatment for the disease. Consequently, the mortality rate of the disease is more than 50% in immunodeficiency patients (Narita et al., 2008).

*Prototheca zopfii* is one of the most common species responsible for human protothecosis, and can be subdivided into genotypes I and II, but only genotype II is pathogenic to humans. It can form creamy-white, yeast-like colonies in Sabouraud dextrose agar after 48 h of incubation at both 37 and 26°C. *P. zopfii* 18125 was isolated from the world’s first case of lymphogranuloma infection by this organism, and has been identified as genotype I by MALDI-TOF MS, which is the first pathogenic genotype I strain of *P. zopfiii. P. zopfii* 50779 was isolated from a meningitis patient and was first reported by us, and has the characteristics of genotypes I and II by MALDI-TOF MS study (Zhang et al., 2007; Hirose et al., 2018). *P. zopfii N71* was isolated from a stream in Japan. Most studies on *Prototheca* have been on isolation and classification of the organism, with very few studies on pathogenicity and biological characteristics. To gain an insight into the pathogenesis of protothecosis, and to tentatively explore some possible virulence genes of the organism, we explored the genetic differences between pathogenic and environmental strains of *P. zopfii* using next-generation sequencing methods.

## Materials and Methods

### Cultivation of *Prototheca zopfii*

The strains used in this article are listed in Table 1. Each strain was cultured in Sabouraud dextrose liquid medium at 37°C to a concentration of OD600 (mid-logarithmic growth phase). Three biological replicates of each strain were grown. We used centrifugation tubes to collect *P. zopfii* and washed the cells twice with phosphate-buffered saline.

**Table 1:** *Prototheca zopfii* strains used in the study.

Name of strain	Description	Pathogenic classification	Source
*P. zopfii*, 18125	*Prototheca zopfii genotype 1*	Pathogen	Lymph tissues of a Granulomatous lymphadenitis patient (Zhang et al., 2010)
*P. zopfii*, 50779	*Prototheca zopfii genotype 2*	Pathogen	Cerebrospinal fluid (CSF) of a meningitis patient (Zhang et al., 2007)
*P. zopfii*, N71	*Prototheca zopfii genotype 1*	Environment	Steam river near a cow barn in Japan (Osumi et al., 2008)


### Genome Sequencing and Assembly

#### DNA Preparation

Genomic DNA of *P. zopfii* 18125, 50779 and N71 was extracted with Qiagen Plant mini kit^®^ following the manufacturer’s protocol. The harvested DNA was detected by agarose gel electrophoresis and quantified by Qubit.

#### *De novo* Genome Sequencing of *P. zopfii* 18125

The genome of strain *P. zopfii 18125* was sequenced by single molecule, real-time (SMRT) technology at the Beijing Novogene Bioinformatics Technology, Co., Ltd. Low quality reads were filtered by SMRT 2.3.0 (Konstantin et al., 2015; Sergey and Adam, 2015), and the filtered reads were assembled to generate one contig without gaps. We used the Augustus 2.7 program to retrieve the related coding gene (Mario et al., 2008). For gene function prediction, we used GO (Gene Ontology) (Ashburner et al., 2000), KEGG (Kyoto Encyclopedia of Genes and Genomes) (Kanehisa et al., 2004), COG (Clusters of Orthologous Groups) (Tatusov et al., 2003), NR (Non-Redundant Protein Database databases) (Li et al., 2002), TCDB (Transporter Classification Database) (Milton et al., 2014), Swiss-Prot (Amos and Rolf, 2000), and TrEMBL (Magrane and UniProt Consortium, 2011) databases. For pathogenicity and drug resistance analyses, we used the PHI (Pathogen Host Interactions) (Urban et al., 2015), CAZy (Carbohydrate-Active enZYmes Database) (Cantarel et al., 2009) to perform pathogen analyses. A whole genome Blast search (E-value less than 1e-5, minimal two alignment length percentage larger than 40%) was performed against each of the above databases (Altschul et al., 1990). Secretory proteins were predicted by the SignalP and TMHMM (Petersen et al., 2011).

#### Genome Sequencing of *P. zopfii* 50779 and *P. zopfii* N71

The genomes of strains *P. zopfii* *N71* and *50779* were sequenced by Illumina MPS (massively parallel sequencing) technology. We built a paired-end DNA library with an insert size of 350 bp, which was sequenced using Illumina PE150 strategy. The sequenced data were filtered to remove low quality data (40 bp base quality lower than 40), reads containing more than 10 bp N, sequence of adapter contaminate (overlap > 15 bp), and duplication contamination. The clean data was used for subsequent analysis (Krueger et al., 2012). We used BWA (v 0.7.8) software to map the clean data to the genome of 18125 (Li and Durbin, 2009). Then, we used SAMTOOLS (v 0.1.18) software to calculate the coverage of the reference sequence to the clean data and make explanations of the alignment results (Li et al., 2009).

#### SNP/InDel Analysis

SNP (single nucleotide polymorphism) mainly refers to the DNA sequence polymorphism caused by single nucleotide variation at the genome level, including transition, transversion, etc. InDel refers to the insertion and deletion of small fragments in the genome. SAMTOOLS (v0.1.18) software (mpileup –m 2 –F 0.002 –d 10000 –u –L 10000) was used for the detection of individual SNPs and insertion and deletion of small fragments (<50 bp), and to analyze the variation of SNP/InDel in the functional regions of the genome (Li et al., 2009).

#### SV Analysis

SV (structural variation) refers to the insertion, deletion, inversion and translocation of the large segments in the genome. We used BreakDancer (v 1.4.4) software to find all those SV differences between the genomes (Chen et al., 2009).

### Transcriptome Sequencing and Assembly

#### RNA Preparation

We collected whole RNA of *P. zopfii* by Trizol^®^ (Invitrogen, 15596026) method. RNA degradation and contamination was monitored on 1% agarose gels. RNA purity was checked using the NanoPhotometer^®^ spectrophotometer (Implen, Westlake Village, CA, United States). RNA concentration was measured by Qubit^®^ RNA Assay Kit in Qubit^®^2.0 Fluorometer (Life Technologies, Foster City, CA, United States). RNA integrity was assessed using the RNA Nano 6000 Assay Kit of the Bioanalyzer 2100 system (Agilent Technologies, Santa Clara, CA, United States).

#### Library Preparation for Transcriptome Sequencing

For RNA sample preparations, 3 μg of RNA per sample were used as input material. Sequencing libraries were generated using NEBNext^®^ UltraTM RNA Library Prep Kit for Illumina^®^ (NEB, United States) following manufacturer’s recommendations, and index codes were added to attribute sequences to each sample. For each strain, we created three biological replicate libraries.

#### Clustering and Sequencing

The clustering of the index-coded samples was performed on acBot Cluster Generation System using TruSeq PE Cluster Kit v3-cBot-HS (Illumina) according to the manufacturer’s instructions. After cluster generation, the library preparations were sequenced on an Illumina Hiseq 2500 platform and 150 bp paired-end reads were generated.

#### Data Analysis

##### Quality control

Raw data (raw reads) of fastq format were firstly processed through in-house perl scripts. Clean data (clean reads) were obtained by removing low quality reads, reads containing adapter, and reads containing poly-N from raw data in this step. At the same time, Q20, Q30, and GC content of the clean data were calculated. These Clean data were the base of all the downstream analyses.

##### Reads mapping to the reference genome

We used the genome of *p. zopfii* 18125 which we mentioned before as the reference genome. Index of the reference genome was built using Hisat2 (v2.0.5), and paired-end clean reads were aligned to the reference genome using Hisat2 (v2.0.5) (Kim et al., 2015). We selected Hisat2 as the mapping tool of this program can generate a database of splice junctions based on the gene model annotation file, and thus a better mapping result than other non-splice mapping tools.

##### Novel transcripts prediction

The mapped reads of each sample were assembled by StringTie (v1.3.3b) (Pertea et al., 2015, 2016) in a reference-based approach. StringTie can assemble and quantitate full length transcripts representing multiple splice variants for each gene locus with a novel network flow algorithm as well as an optional *de novo* assembly step.

##### Quantification of gene expression level

FeatureCounts (v1.5.0-p3) was used to count the reads numbers mapped to each gene (Love et al., 2014). Based on the length of the gene and reads count mapped to this gene, FPKM of each gene was calculated. FPKM, expected number of Fragments Per Kilobase of transcript sequence per Millions base pairs sequenced, considers the effect of sequencing depth and gene length for the reads count at the same time, and is the most commonly used method for estimating gene expression levels currently.

##### Differential expression analysis

Differential expression analysis of two groups (three biological replicates per condition) was done using the DESeq2 R package (v1.16.1) (Love et al., 2014). DESeq2 provides statistical routines for determining differential expression in digital gene expression data using a model based on the negative binomial distribution. The resulting *P*-values were adjusted using the Benjamini and Hochberg’s approach for controlling the false discovery rate. Genes with an adjusted *P*-value < 0.05 by DESeq2, were considered differentially expressed.

##### GO and KEGG enrichment analysis of differentially expressed genes

Gene Ontology (GO) enrichment analysis of differentially expressed genes was performed using the clusterProfiler R package, with gene length bias correction. GO terms with corrected *P*-values of less than 0.05 were considered significantly enriched by differentially expressed genes. KEGG is a database resource for understanding utilities and high-level functions of the biological system, including the cell, organism and the ecosystem, from molecular predicted level information, especially large-scale molecular datasets generated by genome sequencing and other high-throughput experimental technologies^1^. We used cluster Profiler R package to test the statistical enrichment of differential expression genes in KEGG pathways.

### Quantitative Real-Time RT-PCR

#### Primer Design and Synthesis

All the primers were designed by Primer Premier (v6.0.0) software, and the specificity checked by BLAST tool. Details of the primers used are shown in Supplementary Table S1.

#### Preparation of cDNA Library

RNA concentration was measured using Qubit^®^ RNA Assay Kit in Qubit^®^2.0 Flurometer (Life Technologies, Foster City, CA, United States). RNA was reverse-transcribed using PrimeScript^TM^ RT Master Mix (TaKaRa Biotechnology, RR036A) following the manufacturer’s protocol.

#### qRT-PCR Analyses

qRT-PCR analyses were conducted on QuantStudio 3 Real-Time PCR System (Thermo Fisher Applied Biosystems, United States) using TB Green^TM^ Premix Ex Taq^TM^ (TaKaRa Biotechnology, RR420A). The following thermal profile was used: an initial 30 s denaturation step at 95°C, followed by 40 cycles at 95°C for 5 s, and at 60°C for 30 s. Amplification products were analyzed using a 65°C/95°C melting curve. The qPCR raw data were analyzed by QuantStudio software (v 1.4.3), and statistical significance determined using unpaired *t*-test by Holm-Sidak method, with α = 0.05.

## Results

### Genomes of *P. zopfii 18125*, *P. zopfii 50779*, and *P. zopfii N71*

#### *De novo* Genome Sequencing of *P. zopfii* 18125

##### Sequencing assembly

We built a no-gap genome map of strain *P. zopfii 18125* by SMRT technology, which is the first time this has been done. The total reads number was 546,195, with an N_50_ read length of 10,058 bp, and the mean read score was 0.83. Assembly results revealed 56 contigs with a total length of 25,841,422 bp, and the coverage of sequencing was 70X. The key assembly parameters, that is, the largest contig, N_50_, and GC%, were 2,052,191, 1,006,082, and 67.7, respectively.

##### Genome annotations

The IPRscan program was utilized to predict the functional annotation of the identified genes in terms of GO. The largest proportion of genes was associated with metabolic process, cellular process, binding, catalytic activity, cell, and cell part (Supplementary Figure S1). In the KEGG pathway analysis, the largest proportion of genes was associated with translation, carbohydrate metabolism, and amino acid metabolism (Supplementary Figure S2). In addition, we predicted the functional annotation of the identified genes using the KOG database, and the largest proportion of genes was associated with “post-translational modification, protein turnover, chaperones,” and “Translation, ribosomal structure, and biogenesis” (Supplementary Figure S3).

Annotation of the genome information of strain *P. zopfii 18125* in Pfam database revealed “P-loop containing nucleoside triphosphate hydrolase superfamily,” “FAD/NAD(P)-binding Rossmann fold Superfamily,” and “Protein kinase superfamily” and more than 200 related genes (Supplementary Table S2). Through the annotation on NR database, we observed that most genes are found in the genome of *Auxenochlorella protothecoides* and *Helicosporidium* sp. (Supplementary Figure S4). In the TCDB database, we found that the annotated transport proteins in *P. zopfii* were most classified as “P-P-bond-hydrolysis-driven transporters,” “Porters (uniporters, symporters, antiporters),” and “Oxidoreduction-driven transporters” (Supplementary Figure S5).

In PHI database, dozens of genes in *P. zopfii 18125* were annotated. Among these, genes annotated as HSP90 and GroEL have been reported to relate to increased virulence in *Saccharomyces cerevisiae* and *Porphyromonas gingivalis* (Supplementary Table S3).

Details of these results are provided in Supplement Materials.

#### Genome Sequencing of *P. zopfii* 50779 and *P. zopfii* N71

The filtered reads of the genome of *P. zopfii 50779* was 1,697 Mb with a Q20 value 96.3, and that of *P. zopfii N71* was 637 Mb with a Q20 value 96.27. The sequencing depth was 60X in both strains. Compared to the genome of *P. zopfii* 18125, the map rate was 96.45 and 94.73% in *P. zopfii 50779* and *P. zopfii N71*, respectively.

#### SNP/InDel Analysis

**FIGURE 1:**
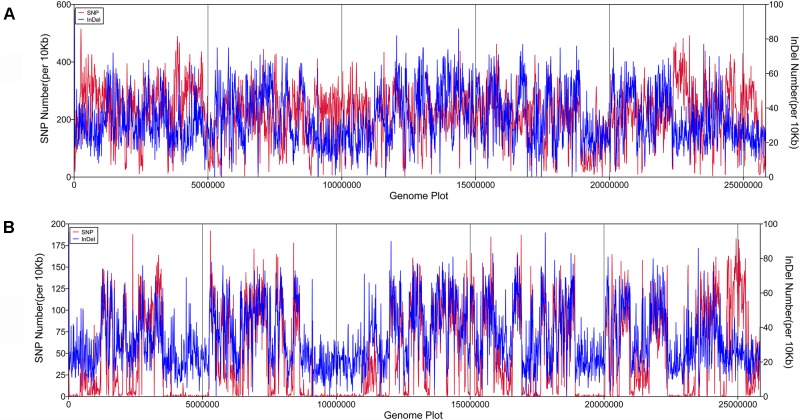
The density and distribution of SNP/lnDel along the reference genome. **(A)** SNP/lnDel distribution in *P. zopfii N71;* **(B)** SNP/lnDel disribution in *Prototheca zopfii 50779.*

SAMTOOLS was used to analyze SNPs and InDels in the genome of *P. zopfii N71* and *P. zopfii 50779*, compared to that of *P. zopfii 18125*. We analyzed the distribution of these variations in the genome as well as the types. After that, an enrichment of those variations was performed in KOG database. In both *P. zopfii N71* and *P. zopfii 50779*, no SNPs or InDels were detected in 5′ UTR region, 3′ UTR region, and Intron region. The number and distribution of SNP/InDel along the genome are shown in Figure 1. The results showed that the number and density of SNP and InDel in *P. zopfii N71* is much larger than *P. zopfii 50779*, and SNP in *P. zopfii N71* shows a significant increase in the regions around 5,000,000 bp, 10,000,000 and 22,500,000 bp in the genome than *P. zopfii 50779*. Especially, the number of SNPs is much larger in *P. zopfii N71* than that of *P. zopfii 50779* in Contig 6. However, the distribution of InDels was similar in *P. zopfii N71* and *P. zopfii 50779*. In the CDS region of *P. zopfii N71* and *P. zopfii 50779*, about 34% SNPs were annotated as non-synonymous mutation and 66% SNPs were annotated as synonymous mutation. The analysis also revealed that more than 80% InDels in the CDS region caused frame-shift in both strains. In enrichment analysis, we found in *P. zopfii 50779* the variation in “Secondary metabolites biosynthesis, transport and catabolism,” “Defense mechanisms,” “Extracellular structures,” and “Nuclear structure” is significant compared with the reference. In *P. zopfii N71* the most significant variation in KOG enrichment was “Translation, ribosomal structure and biogenesis,” “Amino acid transport and metabolism,” “RNA processing and modification,” and “Carbohydrate transport and metabolism.” Details of these findings are shown in Supplementary Table S4.

#### SV Analysis

BreakDancer (v 1.4.4) software was used to find the SVs in the genomes of *P. zopfii 50779* and *P. zopfii N71*. SV number in both *P. zopfii 18125* and *P. zopfii 50779* is similar, and most of it was Interchromosomal migration (CTX), but no insertion SV were found in neither genome. More than 70% of SVs in both strains have a length of 300–400 bp, there is also about 7% of SVs that are more than 1000 bp. There is also 1 ITS and 12 CTS that are similar in the genome of both strains compared to the reference genome. Details of these results are provided in Supplementary Table S5.

### Transcriptomes of *P. zopfii* 18125, *P. zopfii* 50779, and *P. zopfii* N71

#### Assembly of Transcriptome

For each strain, we built three biological replicates. We got about 9G data for each sample with a Q20 above 96%, Q30 value above 90%, and error rate of 0.03, using HISAT program (Supplementary Table S6). Then, we compared the transcriptome data with genome data of strain *P. zopfii 18125*, and found in each of the transcriptomes, above 70% of genes could find a single location in the reference, and genes with multiple map location were lower than 4.3%. Details of these results are provided in Supplementary Table S6.

#### Quantitative Analysis of Transcriptome

In this study, we used feature counts package of the R program to carry out a quantitative analysis of transcriptome, and visualized the results by box and PCA plots. There was an expected correlation among the three biological replicates of each strain, with over 95% correlation (Supplementary Figure S6). Box plot analysis of the results revealed that the overall gene expression levels of the three strains were similar (Supplementary Figure S7). In the PCA plot, biological replicates of each strain were located in the same quadrant, while different strains were located in different quadrants. At the PC1 level, compared with the environment strain, strain *P. zopfii 18125* is located on the left side whilst *P. zopfii 50779* is on the right side. At PC2 level, both pathogenic strains are located on the upside of the environment strain (Supplementary Figure S8).

#### Gene Differential Analysis Results

**FIGURE 2:**
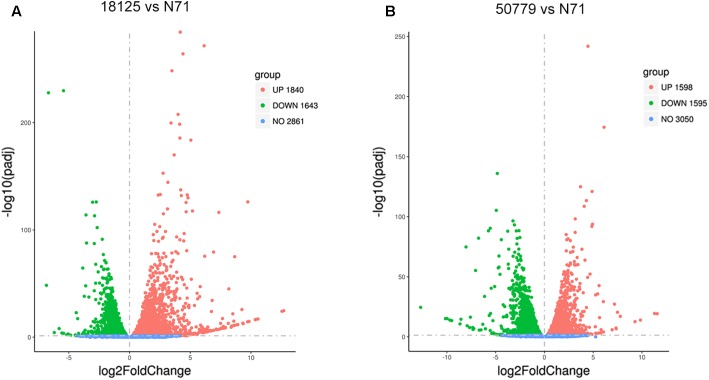
Volcano map shows the distribution of gene expresion difference. **(A)** Volcano map shows all the differential genes between 18125 and N71 in transcriptome. **(B)** Volcano map shows all the differential genes between 18125 and 50779 in transcriptome.

**FIGURE 3:**
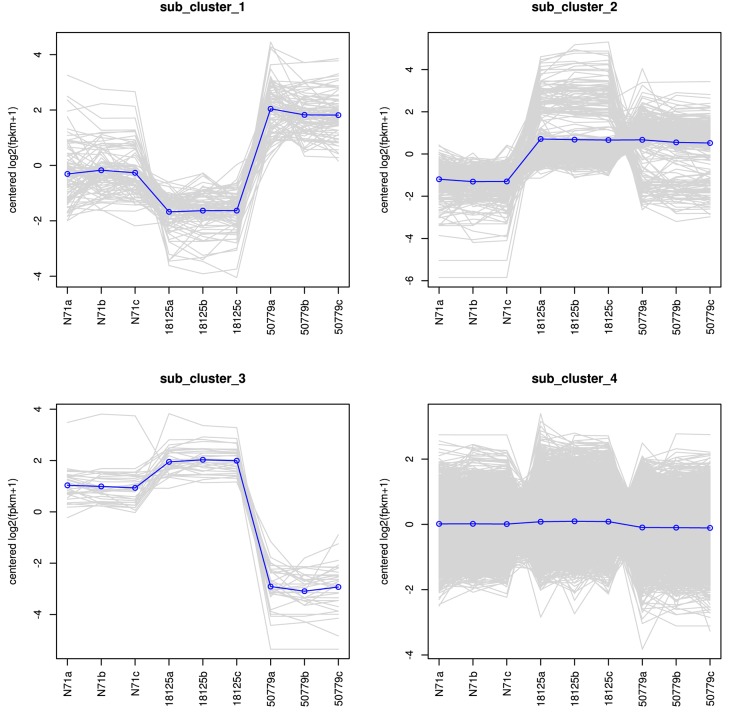
Sub-cluster analysis result of all the differential expression genes in the transcriptome.

The DESeq2 package of R software is used for differential gene expression analysis in nine transcriptomes. Differential expression analysis involves taking the normalized read count data and performing statistical analysis to discover quantitative changes in expression levels between study groups. Using this package, we first compared the gene expression in pathogenic strains vs. environment strain, and visualized the results by volcano plot (Figure 2). There were 3193 significant differential expression of genes in the group *P. zopfii 50779* vs. *P. zopfii N71*, which contained 1598 up-regulated genes and 1595 down-regulated genes, and 3483 significant differential expression of genes in the group *P. zopfii 18125* vs. *P. zopfii N71* that contained 1840 up-regulated genes and 1643 down-regulated genes (Supplementary Table S7). After this, we performed a cluster analysis by H-cluster method, and visualized our results by heat map (Supplementary Figure S9). Cluster analysis involves grouping a set of characteristics in such a way that objects in the same group (a cluster) are more similar (in some way) to each other than those in other groups. We found out that the differential genes were similar in the biological replicates of each strain, and quite different among different strains. We further analyzed these genes by performing a sub-cluster analysis, and the results showed that genes in sub-cluster 2 for pathogen strains were up-regulated compared with those of the environment strain (Figure 3). We used a Venn plot to visualize all the common differential genes we detected in both groups (Supplementary Figure S10), and listed top 10 up- and down-regulated genes in both pathogenic strains which have similar up-regulation level, in Table 2. A comparative analysis was also performed for *P. zopfii 18125* vs. *P. zopfii 50779* as a contrast.

**Table 2:** Top 10 up-regulated and down-regulated genes between two pathogenic strains with environmental strain.

Gene ID	########	N71	log2Fold Change	P value adjustment	Gene_length	Gene description
novel.4689	42.474	0.387	6.707	0.000	3242	–
novel.4682	4420.081	62.465	6.144	0.000	2796	PF00083:Sugar (and other) transporter
WLZA4507	46.809	0.731	5.950	0.000	1197	{NA}
novel.1253	57.094	2.697	4.426	0.000	3982	PF05686:Glycosyl transferase family 90
novel.1261	240.853	16.960	3.823	0.000	5570	–
novel.5859	42.639	3.022	3.816	0.000	2814	PF00782:Dual specificity phosphatase, catalytic domain
novel.412	666.199	55.793	3.578	0.000	2901	–
WLZA0852	997.851	87.243	3.514	0.000	363	{GO:0015986; ATP synthesis coupled proton transport; biological_process GO:0045263; proton-transporting ATP synthase complex, coupling factor F(o); cellular_component GO:0015078; hydrogen ion transmembrane transporter activity; molecular_function}
WLZA4166	57.908	5.774	3.341	0.000	306	{NA}
novel.165	272.167	29.296	3.220	0.000	3361	PF14226:non-hem dioxygenase in morphine synthesis N-terminal| PF03171:2OG-Fe(II) oxygenase superfamily
novel.12	48.130	201.480	–2.064	0.000	6995	PF01423:LSM domain
novel.1369	97.086	411.979	–2.083	0.000	1066	–
novel.2007	130.284	562.728	–2.114	0.000	2309	PF04719:hTAFII28-like protein conserved region
WLZA3571	40.770	185.070	–2.178	0.000	903	{GO:0005643; nuclear pore; cellular_component GO:0003779; actin binding; molecular_function GO:0007010; cytoskeleton organization; biological_process}
novel.879	107.830	532.763	–2.305	0.000	2663	–
WLZA0266	236.856	1177.631	–2.314	0.000	1632	{gi| 633912251| gb| KDD76223.1|; hypothetical protein [*Helicosporidium* sp. ATCC 50920, H632_c309p1]}
novel.5394	107.588	615.300	–2.515	0.000	1929	–
WLZA1865	199.979	1375.247	–2.782	0.000	231	{NA}
WLZA3596	60.329	416.350	–2.784	0.000	261	{gi| 675355111| gb| KFM27551.1|; 50S ribosomal protein L30 [*Auxenochlorella protothecoides*]}
WLZA3745	461.473	3808.836	–3.044	0.000	1257	{gi| 552836688| ref| XP_005849212.1|; hypothetical protein [Chlorella variabilis, CHLNCDRAFT_57448]}


*In this table, most up and down regulated genes were found a significant difference between pathogenic strains and environmental strain in this study. Genes with a FPKM value lower than 40 in the up-regulation strains in comparison were filtered. Also, the significant differential genes between two pathogenic strains were filtered. ^########^Pathgenic Strains.*

In both pathogenic strains, genes related to adherence and penetration process of skin tissues are up-regulated in pathogenic *P. zopfii*, such as Novel.90 annotated as Cysteine dioxygenase type I, Novel.5874 annotated as Eukaryotic aspartyl protease, Novel.1890 and Novel.5198 annotated as Aspartyl protease.

Details of differential genes are listed in Supplementary Table S7.

#### Gene Enrichment of Transcriptome

Gene enrichment analysis (functional enrichment analysis) is a statistical method used to identify genes or protein classes that are over-represented in a large set of genes or proteins, and may be associated with disease phenotypes. We used cluster Profiler program to study the enrichment analysis of different genes identified in the differential analysis, and did the annotation with GO and KEGG databases.

**FIGURE 4:**
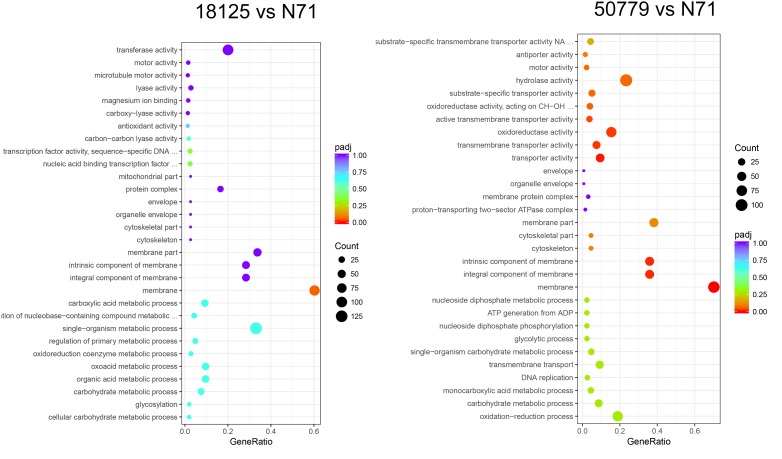
Dot map show the GO enrichment result of up-regulated genes in transcriptome comparation.

**FIGURE 5:**
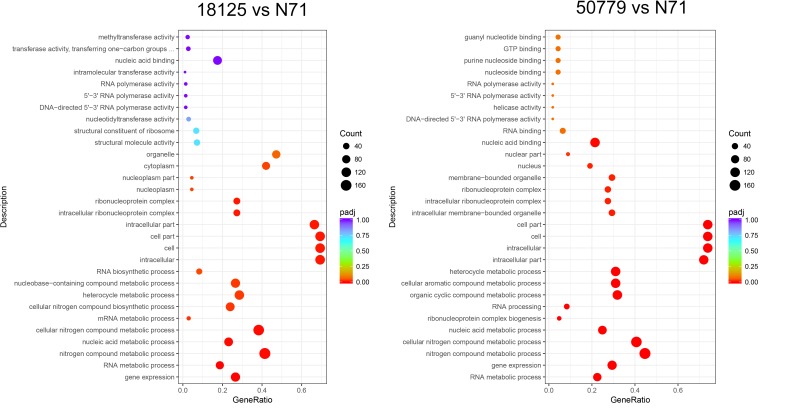
Dot map shows the GO enrichment result of down-regulated genes in transcriptome comparation.

In the GO database, the overall gene expression levels in pathogenic and environment strains were different, but the difference was not statistically significant. So, we analyzed the up-regulated and down-regulated genes separately. In the up-regulated genes, genes related to membrane, integral component of membrane, intrinsic component of membrane, transmembrane transporter activity, oxidoreductase activity, and active transmembrane transporter activity, were significantly up-regulated in the pathogenic strains than environmental ones (Figure 4). Genes related to cell part, cell, intracellular, cellular aromatic compound metabolic process, organic cyclic compound metabolic process, RNA processing, RNA metabolic process, ribonucleoprotein complex biogenesis, and nitrogen compound metabolic process were significantly down-regulated in the pathogenic than environmental strains (Figure 5). We also analyzed the difference between two pathogenic strains as a reference, the result showed that there is no statistical significance in up-regulated genes in two pathogenic strains, but down-regulated genes related to RNA metabolic process, ribonucleoprotein complex biogenesis, rRNA processing, rRNA metabolic process, ribosome biogenesis, nucleic acid binding, RNA binding were statistical significant different (Supplementary Figure S11).

**FIGURE 6:**
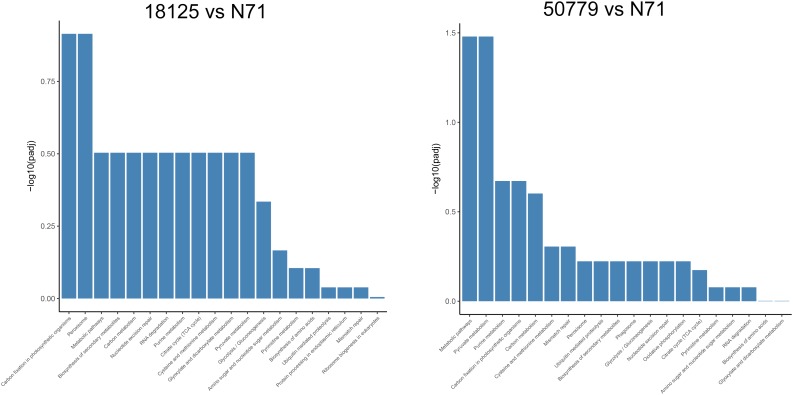
Barmap show the KEGG enrichment result of up-regulated genes in transcriptome comparation.

**FIGURE 7:**
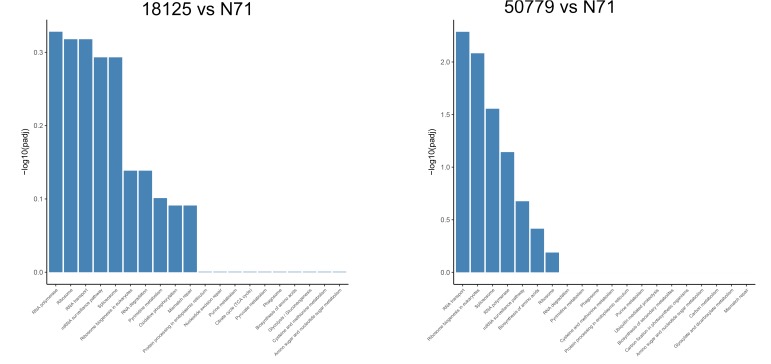
Barmap show the KEGG enrichment result of down-regulated genes in transcriptome comparation.

In the KEGG database, we found that the genes related to carbon fixation in photosynthetic organisms and pyruvate metabolism, were significantly up-regulated in *P. zopfii 50779*. Genes related to carbon fixation in photosynthetic and peroxisome were most up-reregulated in *P. zopfii 18125*, but the adjusted *P*-value was larger than 0.05. Moreover, we found the most up-regulated gene in both pathways mentioned above was WLZ5247, which was annotated as malate dehydrogenase (MDH); also related to glyoxylate cycle (Figure 6). Genes related to RNA transport, ribosome biogenesis in eukaryotes, and spliceosome, were significantly down-regulated in strain *P. zopfii 50779* than the environmental strain. Even though the enrichment of down-regulated genes for strain *P. zopfii 18125* in the KEGG database showed no significant difference with those of the environmental strain, most of these down-regulated genes are also related to the pathways that we mentioned above (Figure 7). Also, genes annotated as PEX12, MPV17, and PRDX5 in peroxisome were up-regulated in *P. zopfii* 18125. An enrichment analysis between two pathogenic strains was also performed, and no statistical significant difference was found (Supplementary Figure S12).

### Quantitative Real-Time RT-PCR Results

We selected some of the most up-regulated and down-regulated genes, including also some other interesting genes, to perform qPCR analysis; 18S RNA was used as endogenous control.

**FIGURE 8:**
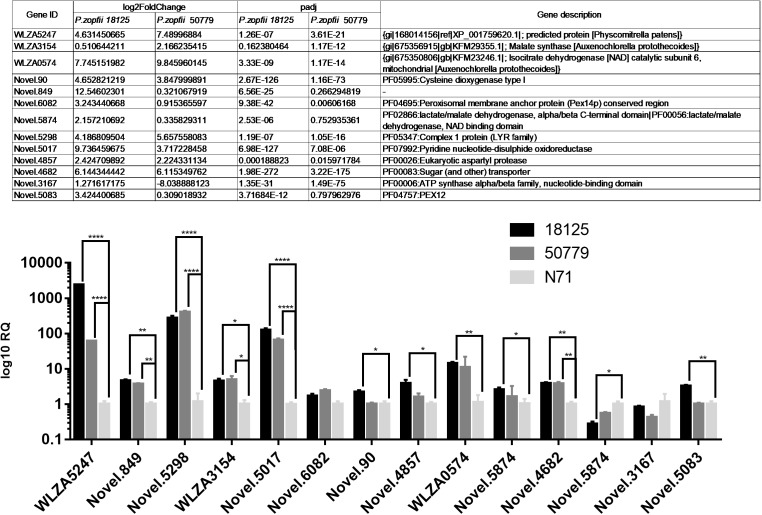
Results of qPCR analysis. Details information about genes in qPCR analysis are in the Table above. Statistical significance determined using the Holm-Sidak method, with alpha = 0.05. Each row was analyzed individually, without assuming a consistent SD. ^∗^*P* < 0.05; ^∗∗^*P* < 0.01; ^∗∗∗^*P* < 0.001; ^∗∗∗∗^*P* < 0.0001.

The qPCR results were in agreement with the results of comparative analysis of transcriptomes as shown in Figure 8. Among these results, gene WLZA5247 which was annotated as MDH, showed a dramatic up-regulation in both pathogenic strains, especially in *P. zopfii 18125*. Novel.5298, which was annotated to code complex 1 LYR protein in Pfam, also showed a high up-regulation. Besides, genes related to glyoxylate cycle such as Novel.90, WLZA3154, and WLZA5874, also showed a dramatic up-regulation. In addition, genes related to known genes related to adherence and penetration process in dermatophytes, such as Novel.90, Novel.4857, also showed a significant up-regulation in pathogenic strains.

Details of these results are provided in Supplementary Table S8.

## Discussion

The increased use of immunosuppressive drugs in routine clinical treatment has resulted in a corresponding increase in the incidence of opportunistic infections, including systemic protothecosis in immunocompromised patients. *Prototheca* is the only known pathogen between that of fungi and plants, and currently research on *Prototheca* is still limited.

Although research on the pathogenesis of protothecosis is scarce, some scholars have studied it. Shahid et al. (2017) found that *P. zopfii* could induce apoptosis in bovine mammary epithelial cells. Murugaiyan et al. (2016), found expression level of Hsp70 protein is significantly up-regulated in *P. zopfii*. Similar results have also been reported by Irrgang et al. (2015), who conducted a proteome study of *P. zopfii GT2, P. zopfii GT1*, and *P. blaschkeae*. It has also been proven that pathogenic *Prototheca* algae can form biofilm which can help it against various environmental changes, and could be related to the pathogenicity (Kwiecinski, 2015). Our group has also published a proteome for a *P. zopfii* strain by iTRAQ technology in 2016, and the results showed that the virulence of *P. zopfii* may be related to suppressed energy production and conversion, carbohydrate transport and metabolism, and in enhanced translation, ribosomal structure, and biogenesis (Liu et al., 2016). However, these previous findings need confirmation in new strains and using a variety of methods. The lack of genome information on the organism has been a big challenge for previous studies on the pathogenesis of *P. zopfii.*

In this study, we constructed the genome and the transcriptomes of two pathogenic strains (18125 and 50779) and one environmental strain (N71), which is the first time this has been done. The results of genome and transcriptome analysis of the studied strains reveal differences and distribution of SNPs, InDels, SVs, and the gene expression differences. Through these results, we found that the genome and transcriptome between pathogenic and environmental strains have some significant difference. In the genome analysis, we found more variations in the *P. zopfii N71* than *P. zopfii 50779* compared to *P. zopfii 18125*. Also, most differential expressed genes among these strains were located in contigs which contained most of these variations. Our findings suggest that genes related to pyruvate metabolism, peroxisome and carbon fixation in photosynthetic organisms, are remarkably up-regulated in pathogenic strains compared to the environmental strain. Also, we found several genes related to secreted aspartyl proteinase which are known protein families related to the virulence of several fungi are significantly up-regulated. We verified these differentially expressed genes by qPCR analysis, and the results were in agreement with those findings. Compared to the previous study, we did not find the up-regulation of HSP70 in either of the pathogenic strains.

Peroxisome is an organelle that extensively exists in eukaryotes, and is vital for many metabolic pathways including β-oxidation and glyoxylate cycle and metabolism of reactive oxygen species (ROS). The function and number of peroxisomes can be regulated through peroxisome life cycle which mainly includes peroxisome biogenesis, peroxisome proliferation, and peroxisome degradation. PEX12 gene codes PEX12 protein that contains two transmembrane domains, and a zinc-binding domain considered to be important for its interaction with other proteins of the peroxisomal protein import machinery (Gootjes et al., 2004). The mutation of this gene will cause synthesis disorder of peroxisome (Sorlin et al., 2016). In our study, Novel.5083 annotated as PEX12 protein family, was significantly up-regulated, as well as several genes related to PEX in pathogenic *P. zopfii*. These results may indicate that peroxisome biogenesis is more active in the pathogenic strains of *P. zopfii*.

Both pathogenic strains showed an up-regulation in both pyruvate metabolism and carbon fixation in photosynthetic organisms compared to the environmental strains in KEGG enrichment. A qPCR analysis was performed to further confirm these results. This result showed that WLZA5247 has a huge up-regulation in pathogenic strains compared with environmental strain, but the up-regulation of other genes related in these pathways were not statistically significant. Also, there is a dramatic mutation of this gene in both pathogenic strains compared with environmental strain. A total of 81 SNPs were found in *P. zopfii N71* compared to *P. zopfii 18125*, while no SNP was detected between two pathogenic strains in this study. Also, additional InDels were detected in *P. zopfii N7*1 vs. *P. zopfii 18125*.

Gene WLZ5247 was annotated as MDH in several databases. Generally, it can generate energy in aerobic conditions through participation in the malate-aspartate shuttle and supplying mitochondria with additional doses of NADH, and transforming oxaloacetate to malate in cytosol. Irrgang et al. (2015) used MALDI-TOF MS to select differential proteins in *P. zopfii* pathogenic strains, and observed that MDH may be a candidate virulence factor. Previous studies also noted that the over-expression of MDH is common in several opportunistic pathogens, including the most common opportunistic pathogen *C. albicans* (Tylicki et al., 2008). Moreover, sera of patients with aspergillosis, infections of *C. albicans*, or *Paracoccidioides brasiliensis*, have been reported to possess antibodies against MDH (Da et al., 2001; Pitarch et al., 2004; Shi et al., 2012).

Besides, MDH can allow fungal cells to use fatty acid as a substrate for gluconeogenesis though glyoxylate cycle (Barelle et al., 2006). The glyoxylate cycle is a modified tricarboxylic acid (TCA) cycle that bypasses the CO_2_-generating steps to conserve carbons as substrates for gluconeogenesis, which consists of the two initial steps of the TCA cycle (catalyzed by citrate synthase and aconitase). Two unique enzymes in glyoxylate cycle isocitrate lyase (ICL) and malate synthase (MS), and malate dehydrogenase (MDH) that connect TCA cycle, have been found to be connected with the virulence of several pathogens. In *Mycobacterium tuberculosis*, down regulation of glyoxylate cycle also leads to a reduced stress tolerance, persistence and survival in macrophages (Singh et al., 2017). This is because phagolysosome is a glucose deficient environment rich in fatty acids or their breakdown products (primarily acetyl-CoA) which makes glyoxylate cycle the only route to the synthesis of glucose that supports the survival of pathogenic fungi in this environment (Lorenz and Fink, 2001).

Also, WLZA3154 was annotated as malate synthase (MS) in *Auxenochlorella protothecoides*, which is a unique enzyme in glyoxylate cycle, and has been reported to be related to the virulence of several pathogenic fungi including *C. albicans* and *Aspergillus fumigatus* (Dunn et al., 2009). These findings highly suggest that glyoxylate cycle may be related to the pathogenesis of protothecosis, and MDH may play an important role in this process.

In addition to genes in the above metabolism pathways, we also found genes related to adherence and penetration process of skin tissues are up-regulated in pathogenic *P. zopfii*, such as gene Novel.90 annotated as cysteine dioxygenase type I (PF05995), Novel.5874 annotated as eukaryotic aspartyl protease (PF00026), and Novel.5198 annotated as aspartyl protease (PF13650).

In dermatophytes, cysteine dioxygenase is crucial for keratin degradation through its involvement in sulfite production (Grumbt et al., 2013). Because keratin is rich in cysteine which is toxic for microbes and humans at elevated concentrations, the cysteine transformation and sulfite efflux pump appear to contribute to cysteine and sulfite tolerance, and to keratin degradation. Sulfite formation from cysteine is due to the effect of the key enzyme cysteine dioxygenase and is supported by the sulfite efflux pump. Also, a hypothesis states that sulfite excreted by the fungus could cleave disulfide bridges which are main stabilizing bonds of keratin (Kunert, 1976), since fungal proteases are not able to hydrolyze compact keratinized tissues unless disulfide bridges are reduced (Monod, 2008). The aspartic proteases secreted by several pathogens are involved in the adherence process and penetration of tissues, and in interactions with the immune system of the infected host. Secreted aspartic proteases of *C.* *albicans* can act as cytolysins being involved in the direct destruction of intracellular components of the macrophages (Monod et al., 2002).

Apart from the up-regulated genes, we also found, through KEGG enrichment study, that genes related to “RNA polymerase,” “Ribosome biogenesis in eukaryotes,” “Spliceosome” were significantly down regulated *in P. zopfii 50779*. Although these pathways were not significantly downregulated in *P. zopfii 18125*, a similar result was also observed. The results of GO enrichment also showed that pathogenic strains had a significant down-regulation in multiple metabolism pathways, such as “cellular nitrogen compound biosynthetic process,” “nucleic acid metabolic process,” “mRNA processing,” and others. These results indicate that the metabolism reactions in pathogenic strains were less active, and metabolism functions in pathogenic strains were declined, which may be related to the degeneration of its organelles (Severgnini et al., 2018). The reduction in metabolism function is common in parasitic protists, and similar gene down regulations were also found in parasitic non-photosynthesizing green alga *Helicosporidium* (Pombert et al., 2014). The loss of chloroplast and reduction in metabolism function may indicate the close relatedness of pathogenic *P. zopfii* to these microorganisms, and may provide insights in understanding the evolution pathway of its pathogenicity.

Because there is no genome or transcriptome for genus *Prototheca* that has been published before, gene annotation is mainly referred to related species, leaving a portion of new genes without an annotation. Thus we could have missed some information because of this. The gene information of a species is very complex, and thus with only three genomes of *P. zopfii* studied, it is difficult to cover all the mutagenesis. Therefore, the genome and transcriptome described in this study does not represent all the characteristics of different *P. zopfii* strains because the variation in strains could be huge. Also, due to limited data of the genome, it is hard to perform a deep analysis to variation in the genome.

## Conclusion

We provide the first genome and the transcriptome of pathogenic strains of *P. zopfii* in humans. Our preliminary findings through bioinformatics analysis show that genes related to pyruvate cycle, glyoxylate cycle and peroxisome metabolism were up-regulated in pathogenic strains of *P. zopfii*, which may improve its ability to survive in glucose deficient environment, and its resistance to the environment in macrophages. Also, we found the up-regulation of several virulence genes related to adherence and penetration process of dermatophytes in pathogenic strains of *P. zopfii*, which may improve its ability in infection processes. Besides, we found genes related to metabolic function were down regulated in pathogenic strains, which may be related to evolution of its pathogenicity. Finally, the genomes and transcriptomes of *P. zopfii* described in this study provide some bases for further studies on the pathogenesis of this organism.

## Biosecurity Statement

All standard biosecurity and institutional safety procedures have been adhered to in all the experiment procedures in this article.

## Data Availability

The BioProject numbers for our study are PRJNA511812 and PRJNA511816^2^. The Biosample numbers in this study are SAMN10639985, SAMN10639986, and SAMN10639987^3^. The SRA numbers for transcriptome data are SRR8447029, SRR8447028, and SRR8447030^4^. The SRA numbers for genome data are SRR8506586, SRR8509414, and SRR8509415^4^.

## Author Contributions

Q-QZ and XZ designed the study and analyzed data. XZ collected samples and conducted the experiments. XZ, TK, and FK discussed, wrote, and finalized the manuscript.

## Conflict of Interest Statement

The authors declare that the research was conducted in the absence of any commercial or financial relationships that could be construed as a potential conflict of interest.

## Footnotes

1 http://www.genome.jp/kegg/

2 https://www.ncbi.nlm.nih.gov/bioproject/

3 https://www.ncbi.nlm.nih.gov/biosample/

4 https://www.ncbi.nlm.nih.gov/sra/

## Supplementary Material

Figure S1 GO annotation of the genome of *P. zopfii 18125.*

Figure S2 KEGG annotation of the genome of *P. zopfii 18125.*

Figure S3 KOG annotation of the genome of *P. zopfii 18125.*

Figure S4 NR annotation of the genome of *P. zopfii 18125*.

Figure S5 TCDB annotation of the genome of *P. zopfii 18125.*

Figure S6 Correlation analysis among samples. R^2^ shows the similarity among biological replication. Generally, R^2^ value more than 0.8 shows good repeatability in biological replication.

Figure S7 Box plot shows the gene distribution in transcriptomes.

Figure S8 PCA plot of the transcriptomes.

Figure S9 Heatmap shows the result of cluster analysis among transcriptomes.

Figure S10 Venn map shows the common differential genes in two groups.

Figure S11 Dot map shows the Go enrichment results between two pathogenic strains. **(A)** Enrichment result of down-regulated genes; **(B)** enrichment result of up-regulated genes.

Figure S12 Bar map shows the KEGG enrichment results between two pathogenic strains. **(A)** Enrichment result of down-regulated genes; **(B)** enrichment result of up-regulated genes.

Table S1 Primer in this study.

Table S2 Pfam anotation of *P. zopfii* 18125 genome.

Table S3 PHI anotation of *P. zopfii* 18125 genome.

Table S4 SNP InDel analysis results between 18125 and N71.

Table S5 SNP InDel analysis results between 18125 and 50779.

Table S6 SV analysis result.

Table S7 Quaility control results of transcripts.

Table S8 Differential analysis results of transcripts.

Table S9 qPCR results.
