# Supplementary material for: Comparative Genome and Transcriptome Study of the Gene Expression Difference Between Pathogenic and Environmental Strains of Prototheca zopfii
Source: Front Microbiol. 2019 Mar 7;10:443. doi: 10.3389/fmicb.2019.00443 (PMC6416184; doi:10.3389/fmicb.2019.00443)
Supplement: Figure S1 — GO annotation of the genome of P. zopfii 18125. [file Data_Sheet_1.doc]

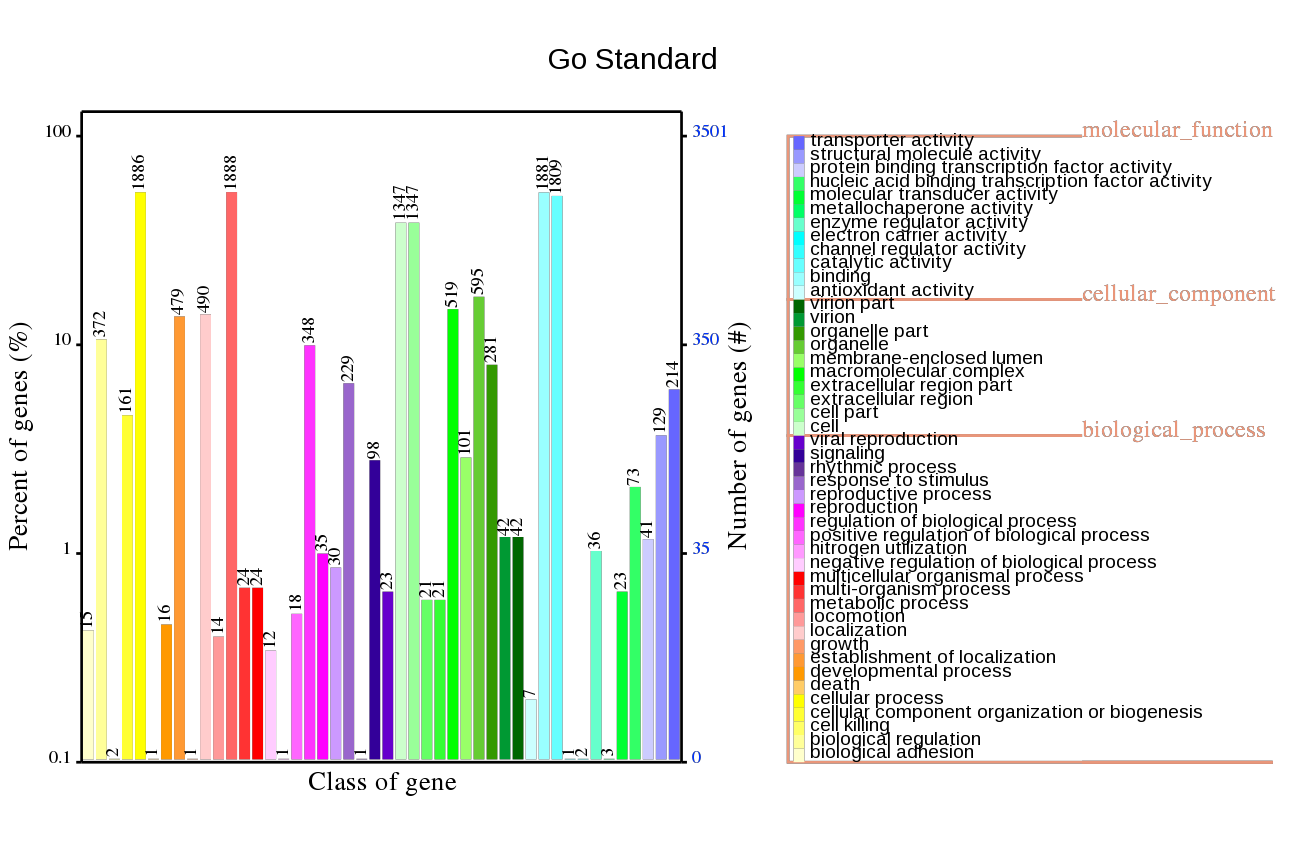
**Supplement Figure.1** GO annotation of the genomo of *P. zopfii 18125*


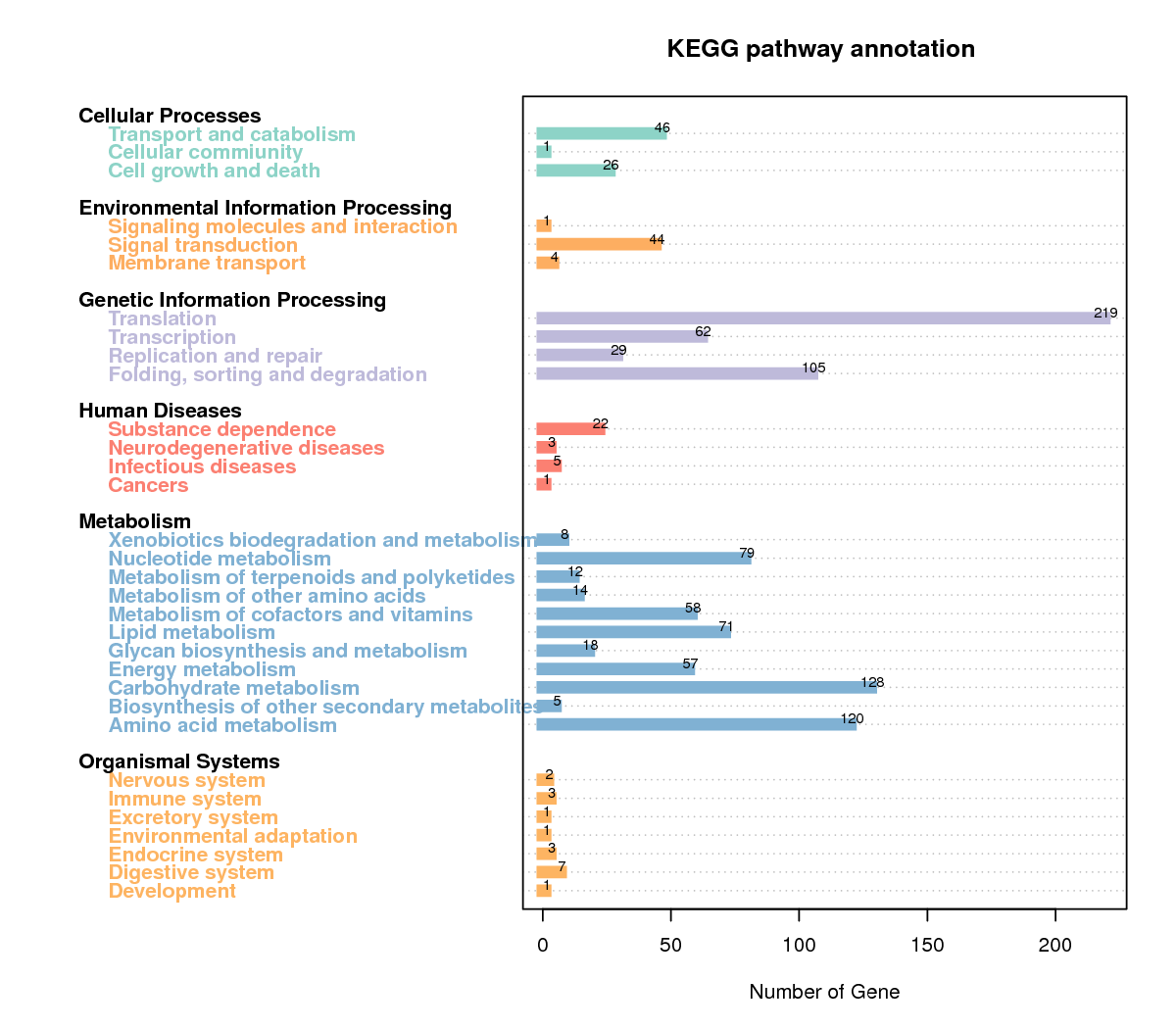


**Supplement Figure.2** KEGG annotation of the genome of *P. zopfii 18125*


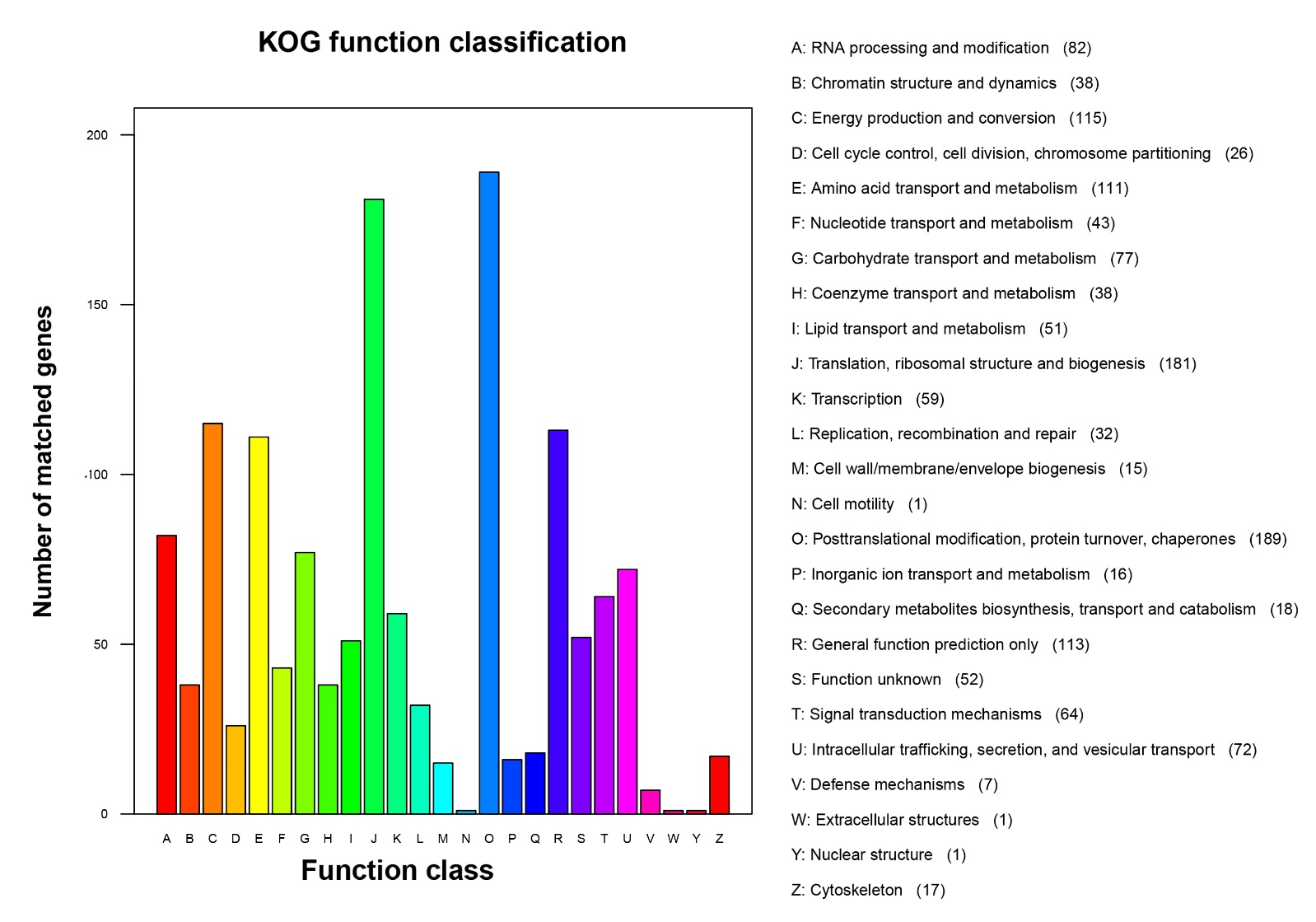
**Supplement Figure.3** KOG annotation of the genome of *P. zopfii 18125*

*
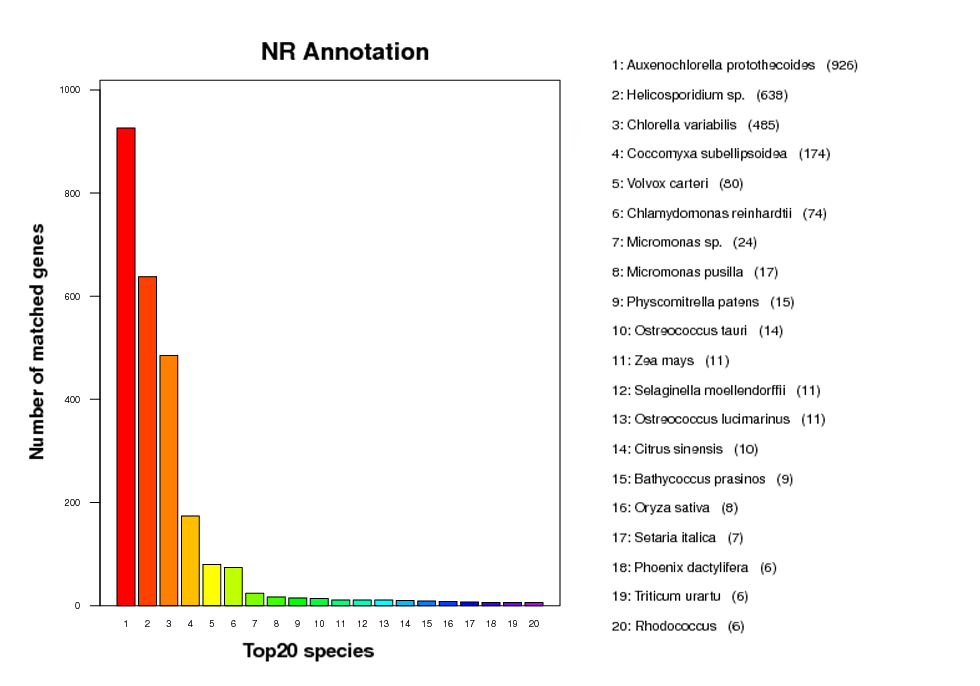
***Supplement Figure.4** NR annotation of the genome of *P. zopfii 18125*

**
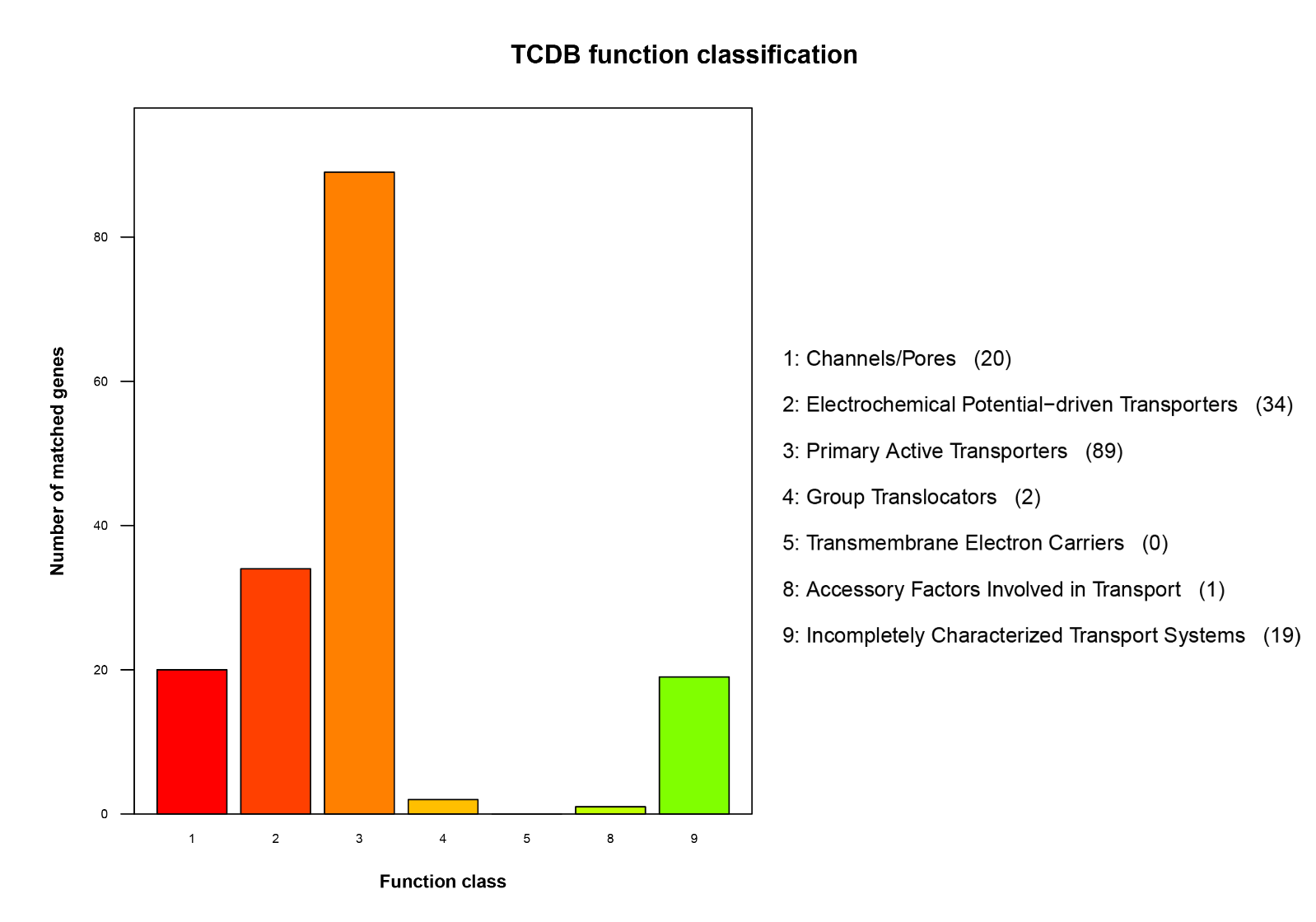
Supplement Figure.5** TCDB annotation of the genome of *P. zopfii 18125*

*
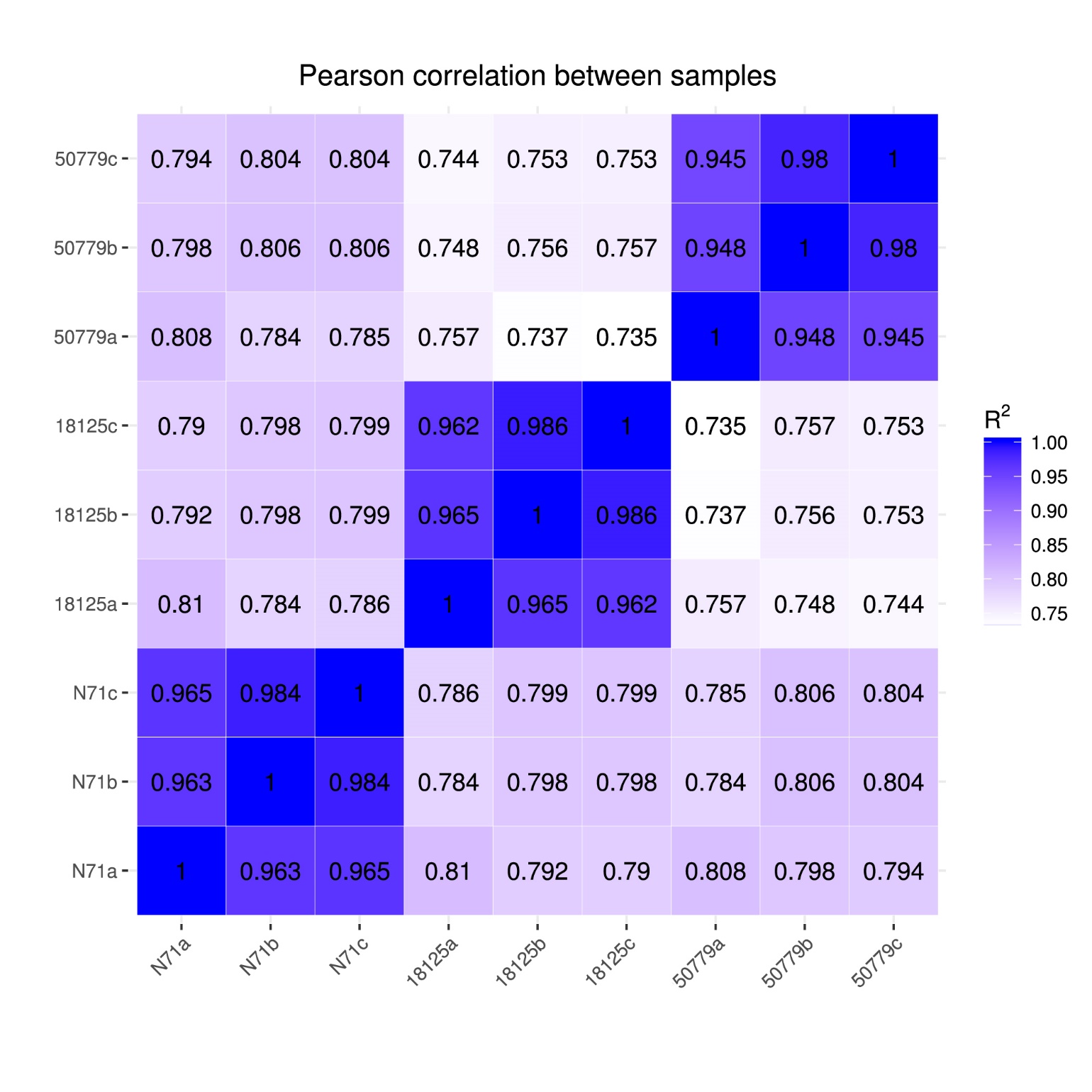
*

**Supplement Figure.6** Correlation analysis among samples. R^2^ shows the similarity among biological replication. Generally, R^2^ value more than 0.8 shows good repeatability in biological replication.

**
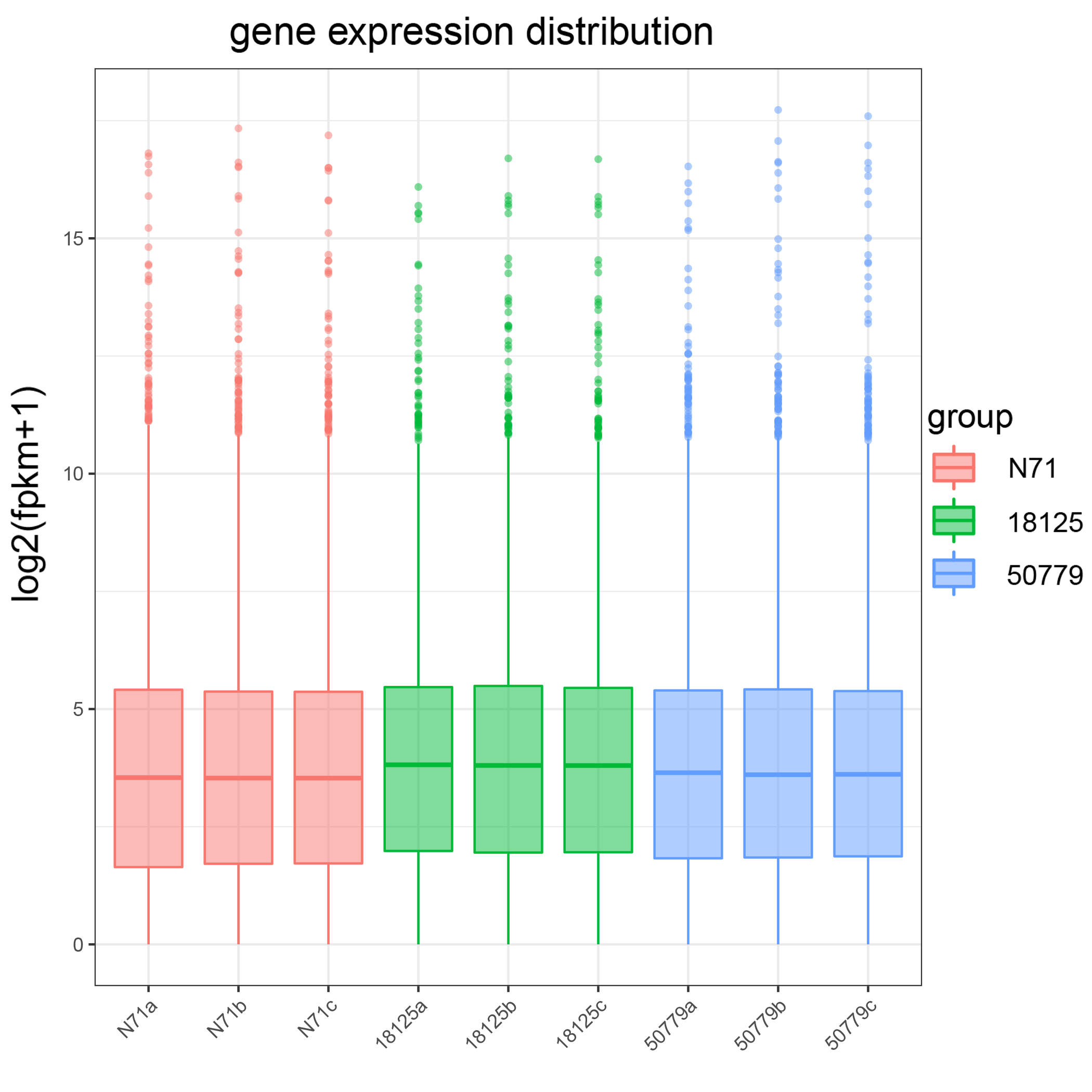
**

**Supplement Figure.7** Box plot shows the gene distribution in transcriptomes

**
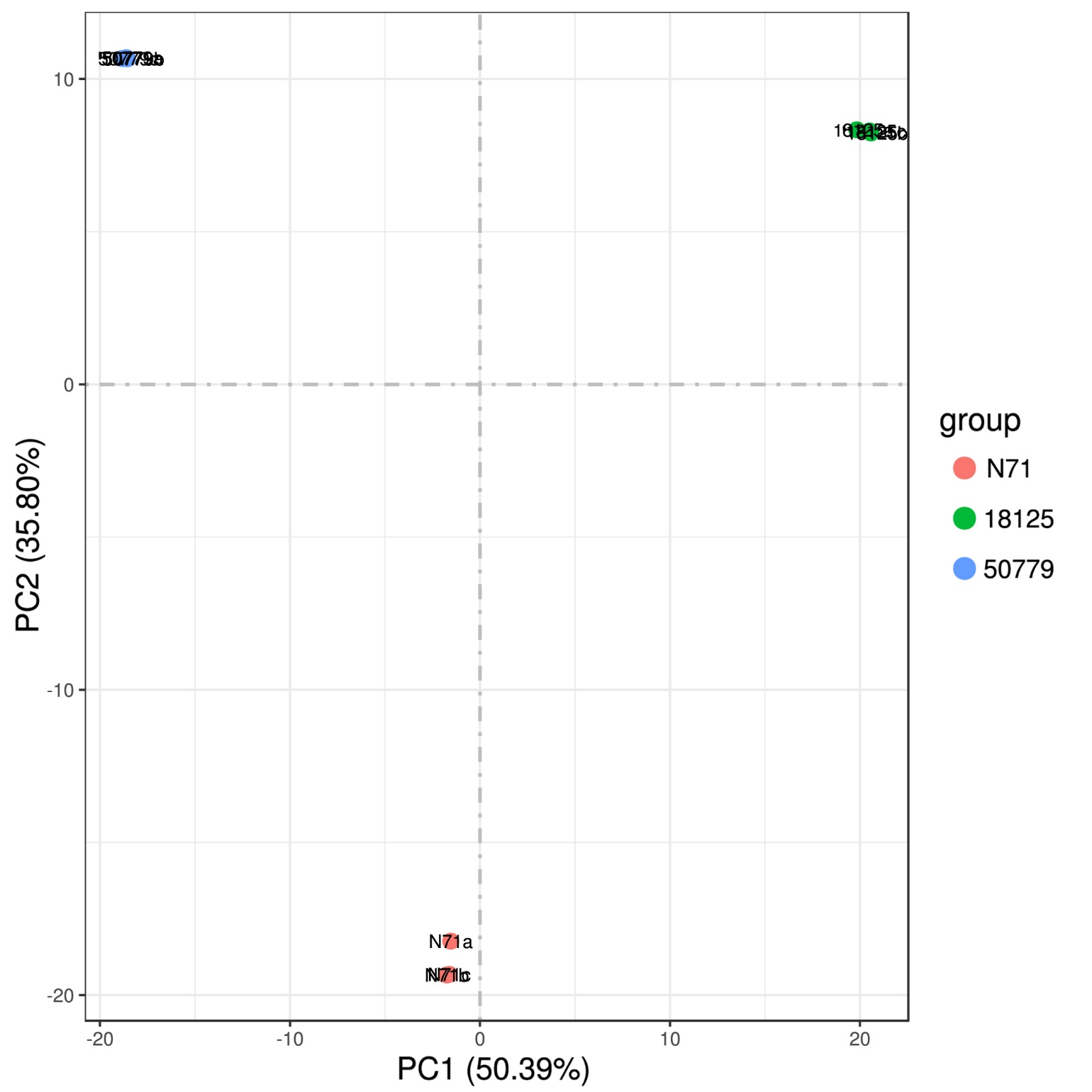
**

**Supplement Figure.8** PCA plot of the transcriptomes.


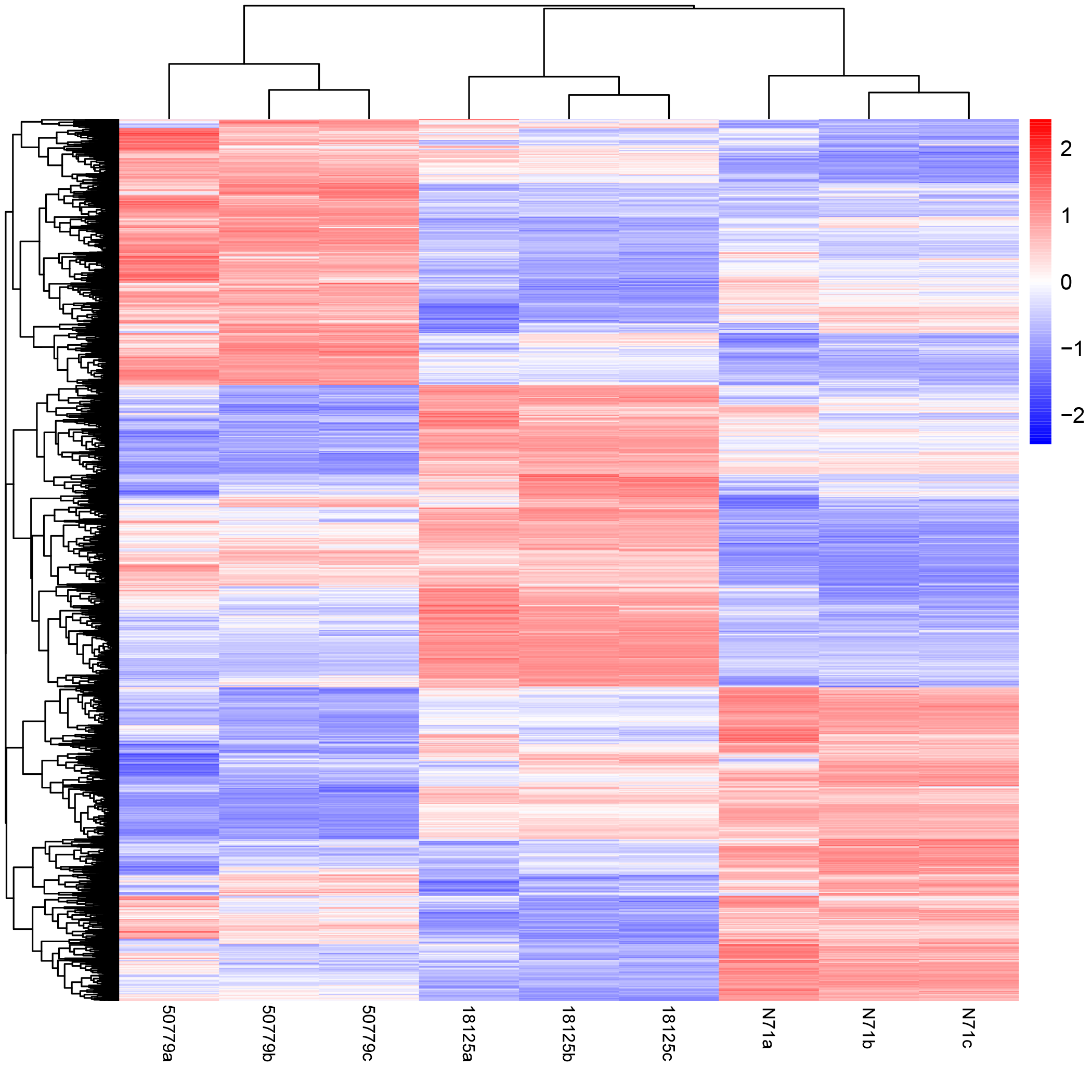


**Supplement Figure. 9** Heatmap shows the result of cluster analysis among transcriptomes.


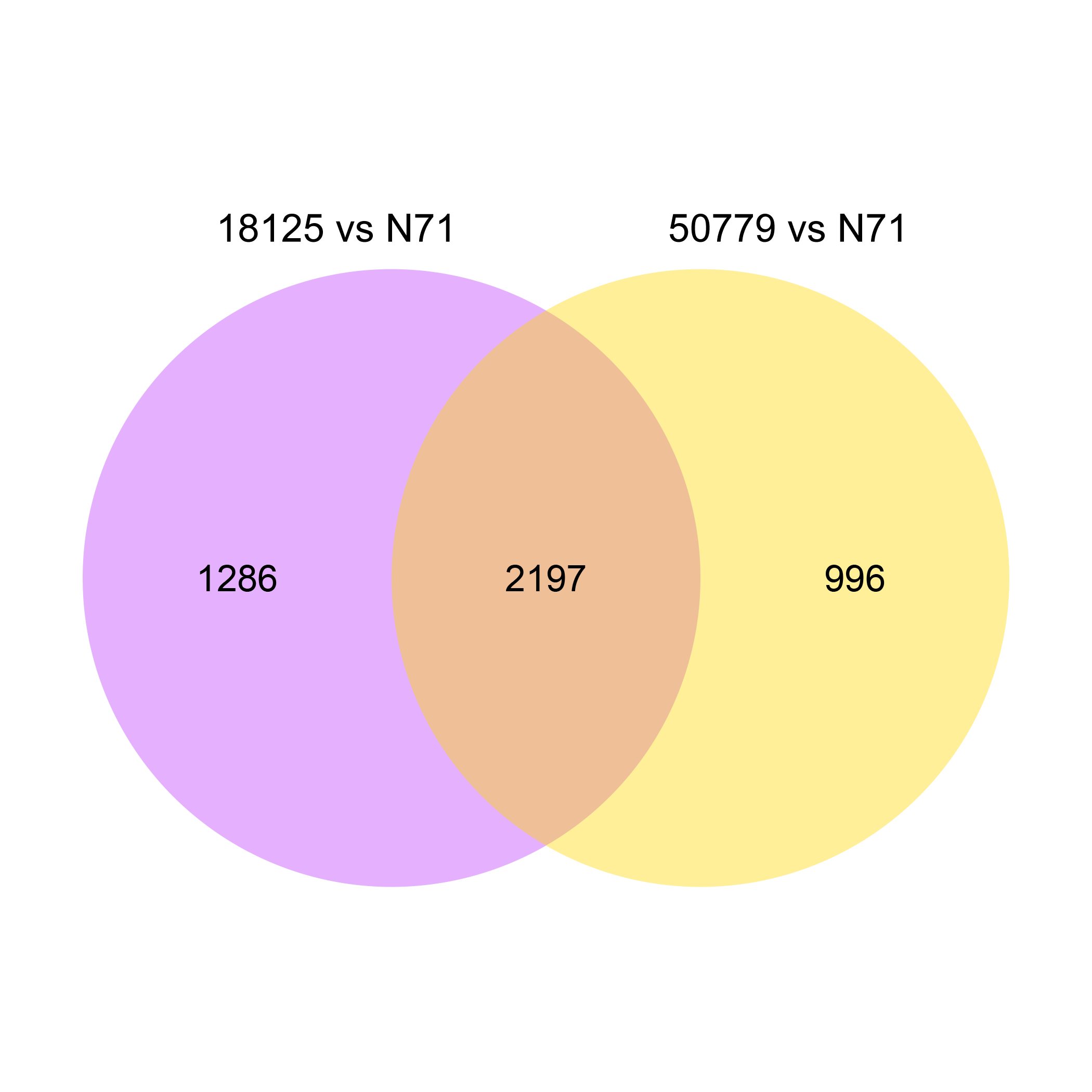


**Supplement Figure.10** Venn map shows the common differential genes in two groups


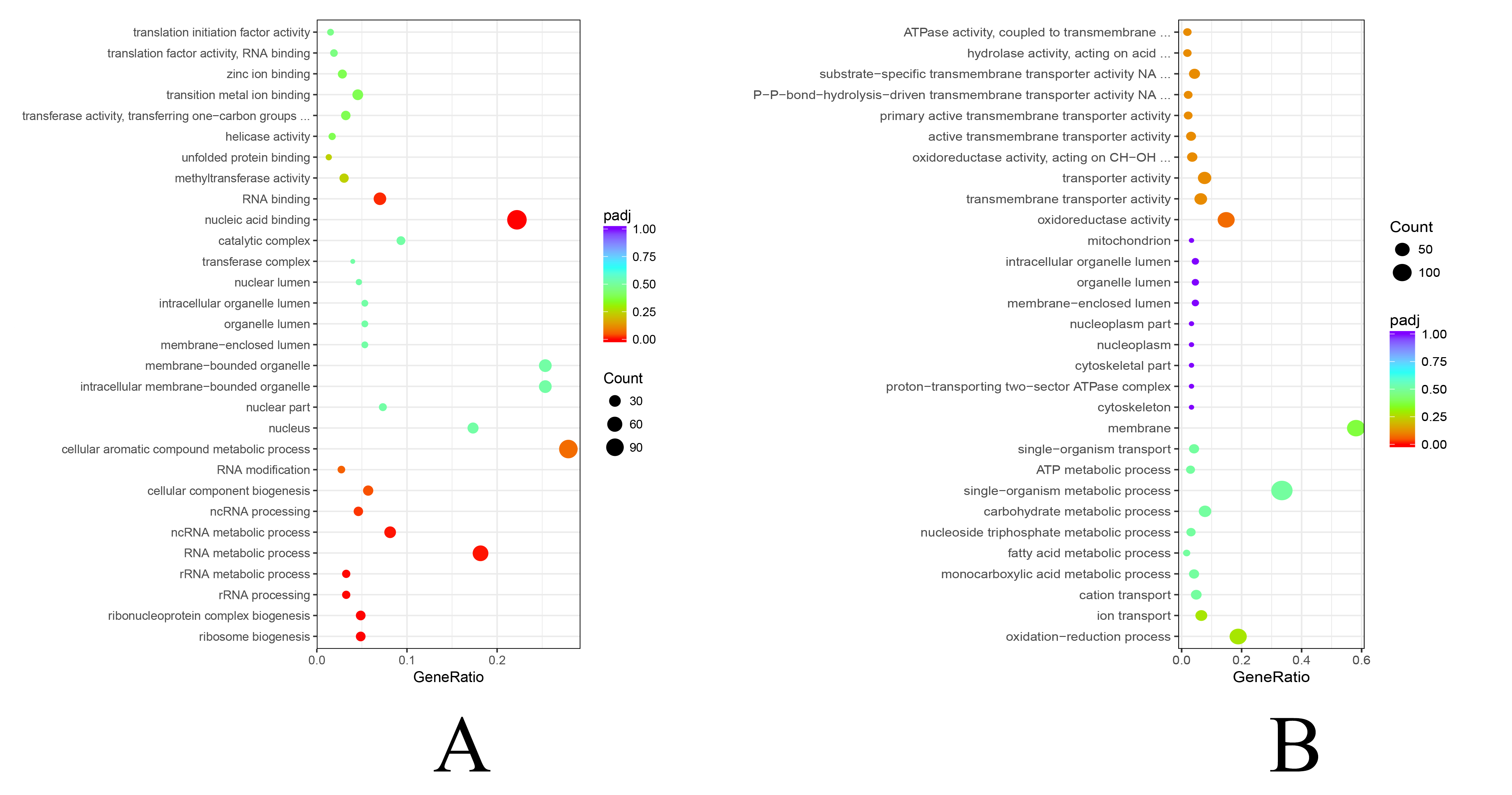


**Supplement Figure.11** Dot map shows the Go enrichment results between two pathogenic strains. A, enrichment result of down-regulated genes; B, enrichment result of up-regulated genes.


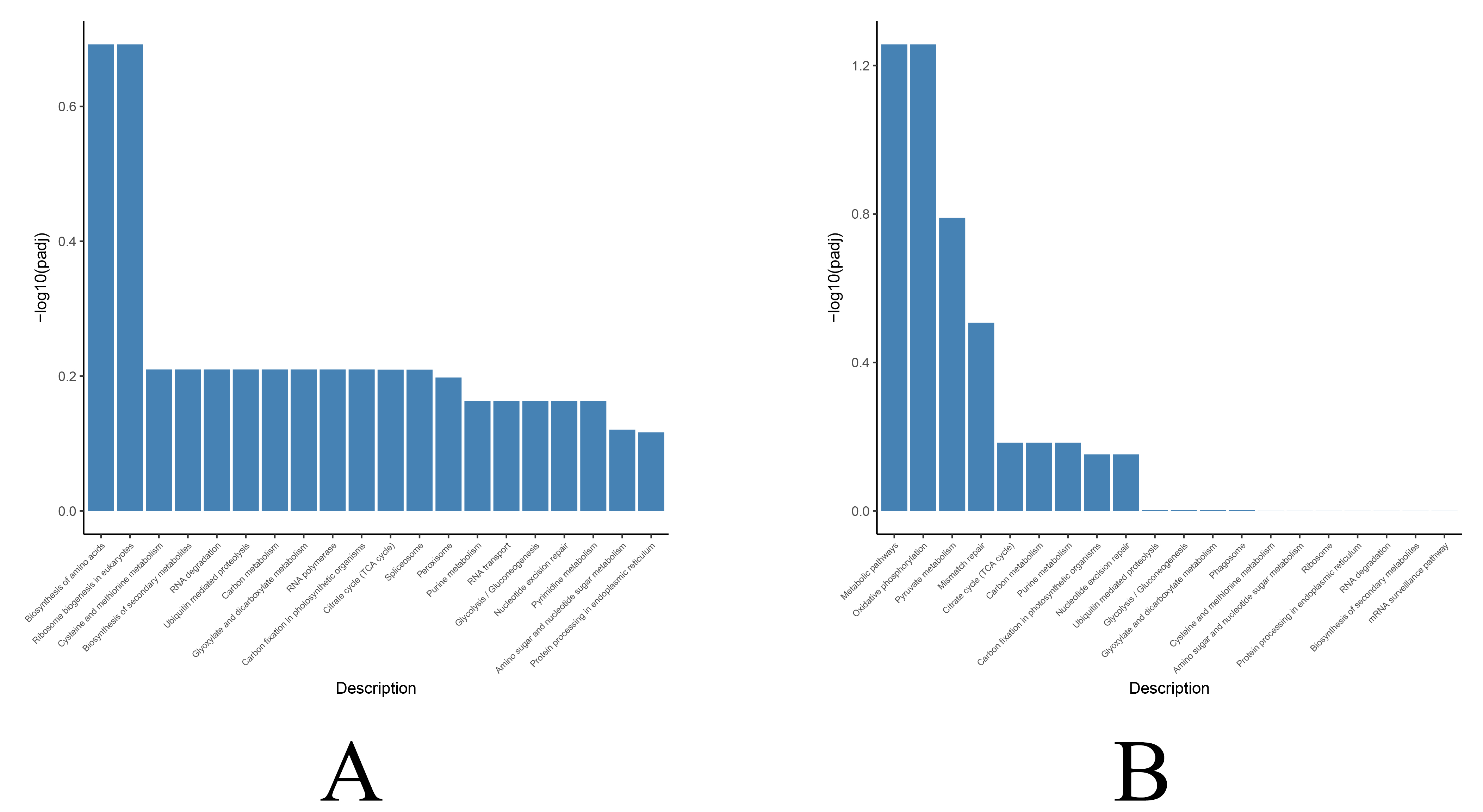


**Supplement Figure.12** Bar map shows the KEGG enrichment results between two pathogenic strains. A, enrichment result of down-regulated genes; B, enrichment result of up-regulated genes.
